
JOURNAL INFORMATION
==============================
NLM Title Abbreviation: Plant Direct
Journal ID: Plant Direct
NLM Journal ID: 101716131
Journal ID: PLD3

Plant Direct

EISSN: 2475-4455
Publisher: Wiley

ARTICLE INFORMATION
==============================
PMCID: PMC13533613
DOI: 10.1002/pld3.70149
Article ID: PLD370149
Article version: 1
Subjects: Issue Information, Issue Information

Issue Information

Electronic publication date: 2026 Sep 1
Publication date: 2026 Sep
Volume: 10
Issue: 9
Electronic Location ID: e70149
Copyright: © 2026 The Authors. Plant Direct published by American Society of Plant Biologists and the Society for Experimental Biology and John Wiley & Sons Ltd.
License: This is an open access article under the terms of the http://creativecommons.org/licenses/by/4.0/ License, which permits use, distribution and reproduction in any medium, provided the original work is properly cited.
License URL: https://creativecommons.org/licenses/by/4.0/

Figure count: 0
Table count: 0
Page count: 1

source-schema-version-number: 2.0
cover-date: September 2026
details-of-publishers-convertor: Converter:WILEY_ML3GV2_TO_JATSPMC version:6.8.6 mode:remove_FC converted:01.09.2026

==============================

Volume 10, Number 9, September 2026

### Plant Direct

Open Access

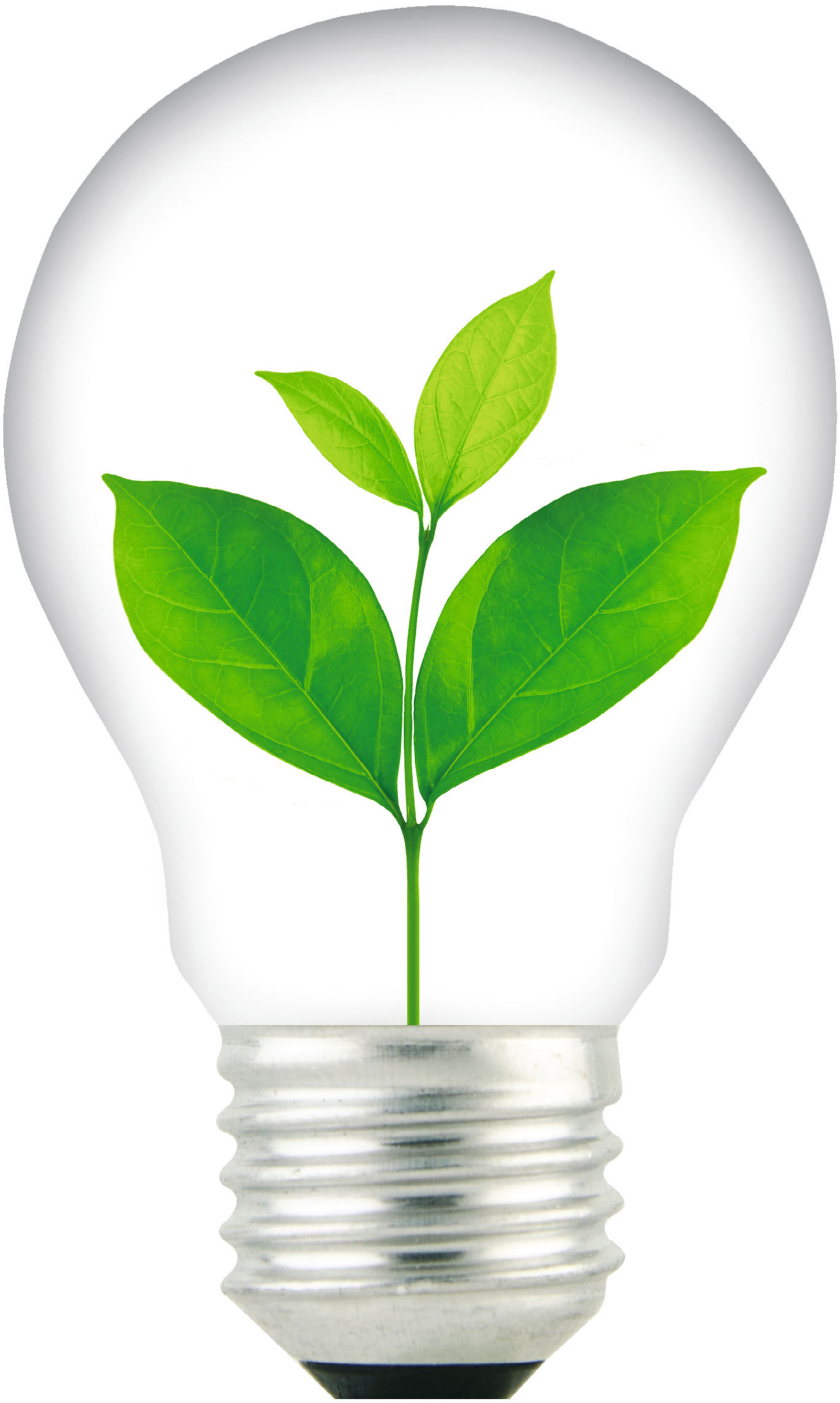

WILEY

American Society of Plant Biologists

Cultivating a better future through plant biology research

SEB

SOCIETY FOR EXPERIMENTAL BIOLOGY

**Editorial Board**

**Editor-in-Chief**

**Richard Haslam,** *Rothamsted Research, Harpenden, UK*

**Supervising Editors**

**Jonathan Ingram,** *John Wiley & Sons, UK*
**Colleen Doherty,** *North Carolina State University, United States*
**Yan Lu,** *Western Michigan University, United States*

**Academic Editors**

**Alexander Schulz,** *University of Copenhagen, Denmark*
**Barbara De Coninck,** *KU Leuven, Belgium*
**Brian Kvitko,** *University of Georgia , USA*
**Chi Zhang,** *University of Nebraska, USA*
**Chin-Mei Lee,** *National Taiwan University, Taiwan*
**Claudia Keitel,** *University of Sydney, Australia*
**Colleen Doherty,** *North Carolina State University, USA*
**David L. Des Marais,** *Harvard, USA*
**David Mendoza-Cozatl,** *Missouri, USA*
**Divya Mishra,** *BRIC-National Institute of Plant Genome Research, India*
**Eva Maria Farre,** *Michigan State University, USA*
**Gaurav Dilip Moghe,** *Cornell University, USA*
**George Littlejohn,** *University of Plymouth, United Kingdom*
**Guillaume Lobet,** *Forschungszentrum Jülich and the Université catholique de Louvain, Germany*
**Hannah, Schneider,** *Wageningen University, the Netherlands*
**Hernâni Gerós,** *Univeristy Minho, Portugal*
**Hongtao Ji,** *Huazhong Agricultural University, China*
**Hsin-Hung Yeh,** *Agricultural Biotechnology Research Center, Academia Sinica, Taiwan*
**Jonathan Fresnedo-Ramírez,** *Ohio State University, USA*
**José M. Seguí-Simarro,** *Universitat Politécnica de Valéncia, Spain*
**Jyothilakshmi Vadassery,** *National Institute of Plant Genome Research, India*
**Kathleen Greenham,** *University of Minnesota, USA*
**Kuo-Chen Yeh,** *Academia Sinica, Taiwan*
**Marco Giovannetti,** *University of Padova, Italy*
**Marna Yandeau-Nelson,** *Iowa State, USA*
**Monica Höfte,** *Ghent University, Belgium*
**Philippe Gallusci,** *INRA, France*
**Reggie Millwood,** *University of Tennessee, USA*
**Rubén Rellán-Álvarez,** *Langebio, Mexico*
**Ryan Sartor,** *North Carolina State University, USA*
**Somnath Koley,** *University of Tennessee, USA*
**Toshiharu Shikanai,** *Kyoto University, Japan*
**Wei Gao,** *Henan University, People's Republic of China*
**Xia Li,** *Huazhong Agricultural University, China*
**Yan Lu,** *Western MI University, USA*
**Ying Gu,** *Penn State, USA*
**Yuki Nakamura,** *Sinica, Taiwan*
**Zhaobo Lang,** *Shanghai Center, China*

**Open Access and Copyright**

All articles accepted and published in *Plant Direct* are fully Open Access: immediately freely available to read, download and share.

Copyright on any research article published by *Plant Direct* is retained by the author(s).

Further information about open access licenses and copyright can be found at: https://authorservices.wiley.com/author-resources/Journal-Authors/licensing/licensing-info-faqs.html#4. Information on the licenses used by *Plant Direct* can be found at: https://plantdirect.org.

**Disclaimer**

The Publisher, *Plant Direct* and Editors cannot be held responsible for any errors in or any consequences arising from the use of information contained in this journal. The views and opinions expressed do not necessarily reflect those of the Publisher, *Plant Direct* or Editors, neither does the publication of advertisements constitute any endorsement by the Publisher, *Plant Direct*, Editors, or Authors of the products advertised.

Wiley Open Access articles posted to repositories or websites are without warranty from Wiley of any kind, either express or implied, including, but not limited to, warranties of merchantability, fitness for a particular purpose, or non-infringement. To the fullest extent permitted by law Wiley disclaims all liability for any loss or damage arising out of, or in connection with, the use of or inability to use the content.

For submission instructions, subscription and all other information visit: https://plantdirect.org.

WILEY
Online Library
